# Effects of Adding a Floss-Band Procedure with Additional Active Shoulder Movement to Conventional Physiotherapy in Selected Patients with Chronic Hemiplegic Shoulder Pain: A Randomized Controlled Trial

**DOI:** 10.3390/jcm15155807

**Published:** 2026-07-24

**Authors:** Ömer Şevgin, Kübra Uzun, Ertuğrul Safran

**Affiliations:** 1 Department of Physiotherapy and Rehabilitation, Faculty of Health Sciences, Üsküdar University, 34768 İstanbul, Türkiye; kubra.uzun3@st.uskudar.edu.tr; 2 Department of Physiotherapy and Rehabilitation, Faculty of Health Sciences, Bezmialem Vakıf University, 34093 İstanbul, Türkiye; esafran@bezmialem.edu.tr

**Keywords:** physiotherapy, range of motion, rehabilitation, sleep quality, stroke

## Abstract

**Background:** Hemiplegic shoulder pain (HSP) can hinder post-stroke rehabilitation. This trial evaluated the incremental effect of adding a floss-band procedure with active shoulder movement to conventional physiotherapy in a selected chronic HSP subgroup. **Methods:** In this randomized trial with blinded goniometric assessment, 40 adults aged 30–55 years, ≥6 months post-stroke, with VAS pain ≥ 3, restricted shoulder motion, ≥90° active flexion and abduction, and Modified Ashworth Scale score 0 were allocated to conventional physiotherapy plus the floss-band procedure (*n* = 20) or physiotherapy alone (*n* = 20), three times weekly for 6 weeks. SPADI and PSQI were co-primary outcomes; VAS and shoulder range of motion were secondary outcomes. SPADI and PSQI were analyzed using group × baseline interaction models with HC3 robust inference; secondary outcomes used baseline-adjusted ANCOVA. Holm correction covered seven outcome-level tests. **Results:** Experimental-minus-control differences favored the experimental group for SPADI at the 25th percentile, median, and 75th percentile of baseline severity (−20.87, −25.32, and −28.34 points) and for PSQI (−2.48, −2.91, and −3.87 points; all *p* < 0.001). Adjusted differences also favored the experimental group for VAS (−2.33), flexion (+32.68°), abduction (+29.13°), internal rotation (+16.35°), and external rotation (+15.95°). Both co-primary joint tests and all five secondary-outcome tests remained significant after Holm correction. The experimental group had worse baseline pain and mobility. No adverse events were observed. **Conclusions:** In this younger, higher-functioning, non-spastic subgroup, the complete experimental procedure produced greater short-term improvements than physiotherapy alone. Because it also included additional movement, treatment time, therapist interaction, and novelty, the specific effect of tissue flossing or band compression cannot be isolated. Larger matched-control trials are required.

## 1. Introduction

Stroke is a leading cause of long-term adult disability and frequently produces an upper motor neuron syndrome that affects the paretic upper limb. Around the shoulder, the combination of muscle weakness, altered muscle tone, and the secondary soft-tissue and capsular changes that follow disrupt the normal mechanics of the glenohumeral complex. Within this context, hemiplegic shoulder pain (HSP) is the most common pain complication after stroke, defined as any subjective pain experienced in the affected or contra lesional shoulder following the event. Reported frequencies vary widely with case definition, timing, and care setting, ranging from roughly a quarter to nearly two-thirds of patients, and a prospective observational cohort recorded a prevalence of about 55% at admission that persisted over the following months [1]. Because it typically emerges during the critical window of upper-limb recovery, HSP is not merely a symptom but an obstacle to rehabilitation itself. The pathophysiology of HSP is heterogeneous and remains incompletely understood. It has been linked to reduced upper-extremity motor function, glenohumeral subluxation, spasticity, capsular restriction, rotator cuff pathology, adhesive capsulitis, complex regional pain (shoulder-hand) syndrome, and central or thalamic pain mechanisms, and contemporary work has argued that these distinct subtypes should be distinguished to allow more targeted physical-therapy management [2,3]. A recurring feature across several of these mechanisms is the loss of normal soft-tissue and fascial mobility around the joint, which contributes to restricted range of motion and to the perpetuation of pain.

The consequences of HSP extend well beyond the shoulder. It limits use of the affected limb, slows motor recovery, prolongs the rehabilitation course, and is associated with anxiety, depressive symptoms, disturbed sleep, and a measurable reduction in health-related quality of life after stroke [1,4]. The interdependence of pain, restricted movement, impaired function, and poor sleep underscores the value of assessing these domains together rather than in isolation when evaluating any new intervention for this population. A broad spectrum of treatments has been applied to HSP, including positioning and supportive slings, conventional therapeutic exercise, electrotherapy, therapeutic taping, intra-articular or subacromial corticosteroid injection, botulinum toxin for the spastic shoulder, and acupuncture. Systematic reviews of these approaches report inconsistent or modest effects and highlight the absence of a single clearly superior strategy, which has sustained interest in simple, low-cost adjuncts that can be added to a standard physiotherapy program [3,5]. Myofascial techniques that target soft-tissue gliding are attractive candidates in this regard. Tissue flossing, or floss-band application, is one such myofascial intervention. Introduced by Starrett and Cordoza, it involves wrapping a latex band circumferentially around a joint or soft-tissue region, typically with approximately 50% overlap and tension, while the patient actively moves the segment under the resulting compression [6,7]. The proposed mechanisms include fascial shearing that restores gliding between tissue layers, a transient vascular occlusion followed by reperfusion of the treated area, and neuromuscular and pain-modulatory effects; however, the responsible mechanisms remain only partly established [7]. In healthy and athletic populations, floss-band application has been associated with changes in joint mobility, muscle function, pain, and performance in experimental studies and has also been evaluated in systematic reviews [8,9,10,11].

Despite this growing body of evidence, the literature on tissue flossing is dominated by healthy participants and athletes and overwhelmingly concerns the lower limb. Direct evidence in neurological populations is scarce. To date, the only randomized controlled study to apply a floss band to stroke patients examined the ankle joint in chronic stroke and reported immediate improvements in dorsiflexion, balance, and gait relative to a sham band [12]. The hemiplegic shoulder, where soft-tissue and fascial restriction may contribute to the clinical presentation, has not been investigated, and the incremental effect of adding a floss-band procedure with additional active shoulder movement to conventional physiotherapy on shoulder range of motion, function, pain, and sleep quality in chronic HSP remains unknown.

Accordingly, this randomized controlled trial evaluated the incremental effect of adding a floss-band procedure with additional active shoulder movement to conventional physiotherapy in selected patients with chronic HSP. The estimand therefore concerned the complete experimental procedure rather than tissue flossing or band compression in isolation. The registered co-primary outcomes were shoulder-related pain and disability measured using SPADI and sleep quality measured using PSQI. Pain intensity and shoulder range of motion were evaluated as secondary outcomes. We hypothesized that conventional physiotherapy plus the complete experimental procedure would produce greater improvement in the co-primary outcomes than conventional physiotherapy alone, with supportive improvements in pain intensity and shoulder mobility.

## 2. Materials and Methods

### 2.1. Trial Design and Registration

This was a single-center, parallel-group, two-arm randomized controlled trial with a 1:1 allocation ratio and blinded goniometric assessment. The control group received conventional physiotherapy alone, whereas the experimental group received the same physiotherapy program plus the floss-band procedure with additional active shoulder movement. Accordingly, the trial evaluated the incremental effect of the complete experimental procedure rather than the isolated physiological effect of band compression.

The ClinicalTrials.gov record was first submitted on 21 November 2025 and first publicly posted on 3 December 2025. Recruitment commenced on 3 December 2025, after public posting of the registry record and before the first participant provided written informed consent or underwent baseline assessment. The trial was registered as NCT07261007, and the complete record is available at https://clinicaltrials.gov/study/NCT07261007 (accessed on 27 June 2026). The study was conducted at the NRL Physical Therapy Center, Sancaktepe, Istanbul, Türkiye.

The registered primary outcomes were SPADI and PSQI, corresponding to the co-primary outcomes in the present report, and the registered eligibility criteria corresponded to those applied during recruitment. The registry outcome fields initially listed an 8-week time frame; however, the detailed registry description, study protocol, intervention schedule, and actual study procedures specified assessment at baseline and immediately after the six-week intervention. No 8-week assessment was conducted. The registry time-frame entry was therefore corrected to week 6.

A standalone prospectively time-stamped statistical analysis plan was not prepared before recruitment, and the registry did not specify the detailed modelling strategy. The baseline-adjusted ANCOVA models, group × baseline interaction models, HC3 robust inference, joint outcome-level testing, Holm correction, diagnostic analyses, and additional sensitivity analyses are described fully in Section 2.8. These analytical refinements did not change the registered co-primary outcomes or the assessment time point. The study is reported in accordance with the CONSORT statement. During the preparation of this manuscript, the authors used a generative artificial intelligence tool solely to improve English language, readability, and clarity. The tool was not used to generate research data, perform statistical analyses, interpret the findings, or formulate scientific conclusions. All AI-assisted content was carefully reviewed and edited by the authors, who take full responsibility for the accuracy and integrity of the manuscript.

### 2.2. Ethics

The study was approved by the Non-Interventional Research Ethics Committee of Üsküdar University (meeting no. 05, 20 May 2025; decision no. 61351342/020-1175) and was conducted in accordance with the Declaration of Helsinki. Written informed consent was obtained from all participants before enrolment and before any study-specific assessment or intervention was performed.

### 2.3. Participants

Forty participants of both sexes, aged 30–55 years, were enrolled. Participants were eligible if they had a clinical diagnosis of stroke, were in the chronic phase of recovery (≥6 months post-stroke), had pain in the affected shoulder with a Visual Analogue Scale (VAS) score of ≥3, and demonstrated clinically restricted shoulder range of motion. Additional eligibility criteria included at least 90° of active shoulder flexion and abduction, stable vital signs, the ability to understand the study aims and procedures, and willingness to provide written informed consent. All randomized participants met both the pain and range-of-motion criteria; no participant was enrolled solely on the basis of restricted shoulder mobility.

Stroke diagnosis and chronicity were established from the participant’s medical history and available clinical records. Pain eligibility was based on participant-reported pain in the affected shoulder, and shoulder mobility was evaluated by clinical examination. Upper-limb spasticity was assessed using the Modified Ashworth Scale, and only participants with a score of 0 were eligible. Participants were excluded if medical records, available imaging, or clinical examination indicated a major orthopedic shoulder disorder, including fracture, dislocation, or a clinically diagnosed rotator cuff tear. No study-specific diagnostic imaging protocol or independent orthopedic adjudication was performed.

Participants were also excluded if they had skin lesions, peripheral circulatory disorders, a known latex allergy, an acute inflammatory condition, or an acute pain episode that could preclude safe floss-band application. Safety screening before enrolment was based on medical history, available clinical records, participant questioning, and clinical inspection. Skin integrity, latex allergy, sensory impairment relevant to compression safety, and peripheral circulatory problems were assessed. Participants were also questioned about diabetes-related skin complications, peripheral vascular disease, anticoagulant use, and other conditions associated with increased bruising, skin injury, or circulatory risk. Individuals for whom floss-band compression was considered clinically unsafe were excluded.

Stroke type, exact time since stroke beyond the ≥6-month eligibility threshold, detailed motor and sensory impairment, glenohumeral subluxation, visuospatial neglect, limb dominance, and specific hemiplegic shoulder pain phenotype were not systematically recorded. Participants were therefore not classified into subluxation-related, adhesive-capsulitis-related, central post-stroke pain, complex regional pain syndrome, or predominantly soft-tissue-related phenotypes. Consequently, the study population represented a selected, relatively young, higher-functioning subgroup with preserved active shoulder elevation and no clinically detectable upper-limb spasticity rather than the broader chronic hemiplegic shoulder pain population.

### 2.4. Randomization and Blinding

After completion of the baseline assessment, participants were allocated in a 1:1 ratio to the experimental or control group using simple unrestricted computer-generated randomization without blocking or stratification; therefore, no block size was applicable. The allocation sequence was generated before enrolment by an independent researcher who had no role in participant recruitment, baseline assessment, treatment delivery, outcome assessment, or statistical analysis. Group assignments were placed in sequentially numbered, opaque, sealed envelopes prepared according to the generated sequence. The envelopes were opened consecutively only after the participant had been enrolled and the baseline assessment had been completed. Following completion of the study, the original randomization sequence and allocation log were independently cross-checked by a researcher who had not participated in recruitment, treatment delivery, outcome assessment, or statistical analysis. All 40 recorded allocations corresponded to the prespecified sequence, and no deviations in assignment or envelope order were identified.

Owing to the physical and readily recognizable nature of the intervention, participants and the treating physiotherapist were aware of treatment allocation. SPADI, PSQI, and VAS were participant-reported outcomes and were therefore obtained from unblinded participants. Blinding was limited to the physiotherapist who performed all goniometric shoulder range-of-motion measurements at both assessment time points. No formal blinding check was conducted, and possible disclosure of treatment allocation to the goniometric assessor was not systematically recorded; therefore, successful maintenance of assessor blinding throughout the trial cannot be confirmed. Treatment credibility and participant expectations were not prospectively assessed. Demographic and baseline clinical characteristics were recorded before allocation was revealed.

### 2.5. Interventions

Both groups received treatment three times weekly for six weeks, corresponding to 18 planned sessions per participant. The conventional physiotherapy component was identical in both groups and lasted approximately 40–50 min per session. In the experimental group, the floss-band procedure with active shoulder movement was administered after completion of the full conventional physiotherapy program and required up to approximately three additional minutes per session. No component of conventional physiotherapy was shortened or omitted to equalize total session duration. Accordingly, the experimental group received up to approximately three minutes more treatment and therapist interaction per session than the control group.

Conventional physiotherapy program in both groups. Each session began with stretching exercises for shoulder flexion, abduction, internal rotation, and external rotation. The stretches were performed as 10 repetitions held for 15 s, with the participant positioned comfortably in supine and the movements applied by the physiotherapist. These were followed by wand exercises performed as two sets of 10 repetitions. Participants then performed isometric exercises against a wall with the elbow positioned at 90° of flexion. Isometric shoulder flexion, extension, and abduction were completed as two sets of 10 repetitions, with each contraction held for five seconds.

Floss-band procedure with additional active shoulder movement in the experimental group. After completing the conventional physiotherapy program, participants in the experimental group received floss-band application around the affected shoulder. The elastic band was applied to compress the shoulder region while the participant actively moved the joint. Wrapping covered the deltoid, pectoralis major, and subscapularis line, beginning at the mid-distal portion of the deltoid and progressing spirally from distal to proximal. With the band in place, participants performed 10 repetitions of active shoulder flexion, extension and abduction for up to approximately three minutes.

The band used was a Sanctband CompreFloss flossing band (green; natural rubber latex; 5 cm in width and 206 cm in length). Tension was standardized using an elongation-based method. The resting band length was marked before application, and the band was stretched to approximately 150% of its resting length, corresponding to approximately 50% elongation-based tension [10,11,12]. Each turn overlapped the preceding layer by approximately 50% of the band width. The experimental procedure was delivered by a single physiotherapist experienced in floss-band application.

The control group received the complete conventional physiotherapy program without floss-band application or the additional active shoulder movements performed under compression. It did not receive sham wrapping, low-tension placebo wrapping, or an attention-, time-, and exercise-matched procedure. Therefore, the between-group contrast represented the incremental effect of the complete experimental procedure, including band compression, additional active shoulder movement, additional treatment time and therapist interaction, intervention novelty, and participant expectations, rather than the isolated physiological effect of tissue flossing or band compression.

Treatment fidelity, attendance, and safety monitoring. Across the 40 participants, 720 participant-sessions were planned: 360 in the experimental group and 360 in the control group. Session logs documented completion of all 720/720 planned sessions (100%), including 360/360 sessions in each group. No session was missed or recorded as incomplete. For all 360 experimental-group sessions, the logs also confirmed delivery of the intended wrapping technique, band tension, active-movement dose, and application duration.

At each experimental-group session, participants were questioned about pain, numbness, tingling, dizziness, and unusual discomfort. The treated area was inspected before, during, and immediately after application for adverse skin or circulatory responses. The procedure was to be discontinued immediately if symptoms increased or if pallor, cyanosis, excessive redness, delayed capillary refill, or another clinically concerning response was observed. Any adverse event was to be documented in the participant’s treatment record.

### 2.6. Primary and Secondary Outcome Measures

The registered co-primary outcomes were the Shoulder Pain and Disability Index (SPADI) and the Pittsburgh Sleep Quality Index (PSQI). Pain intensity assessed using the Visual Analogue Scale (VAS) and shoulder range of motion were treated as secondary outcomes. SPADI, PSQI, and VAS were participant-reported measures completed by participants who were aware of their treatment allocation. These outcomes were therefore not protected by assessor blinding. In contrast, shoulder range of motion was measured goniometrically by a physiotherapist who was intended to remain blinded to group allocation. No treatment-credibility, expectation, or formal assessor-blinding assessment was included in the study protocol. All outcomes were assessed at two time points: before the start of treatment and immediately after completion of the 6-week intervention period.

Co-primary outcome—Shoulder pain and disability. Functionality was evaluated with the Shoulder Pain and Disability Index (SPADI) [13], comprising a total score and two subscales: a 5-item pain subscale and an 8-item disability (activity-limitation) subscale, with responses marked numerically from 0 to 10. The Turkish version of the SPADI has shown adequate validity and reliability [14]. The total score ranges from 0 to 100, with higher scores indicating greater shoulder pain and disability [15].

Co-primary outcome—Sleep quality. Sleep quality was assessed with the Pittsburgh Sleep Quality Index (PSQI) [16], which provides a quantitative measure of sleep quality over the preceding month across components including subjective sleep quality, sleep latency, sleep duration, habitual sleep efficiency, sleep disturbances, use of sleep medication, and daytime dysfunction. The Turkish version of the PSQI has demonstrated adequate validity and reliability [17]. Total scores range from 0 to 21, with scores greater than 5 indicating poor sleep quality. Previously reported estimates of clinically important change vary across populations and estimation methods [18].

Secondary outcome—Pain intensity. Pain was assessed with the Visual Analogue Scale (VAS), on which 0 indicates no pain and 10 indicates unbearable pain [19].

Secondary outcome—Shoulder range of motion. Shoulder range of motion was measured with a universal goniometer for flexion, abduction, and internal and external rotation, with care taken to prevent compensatory movements. For shoulder flexion, the fulcrum was placed over the greater tubercle of the humerus, the stationary arm parallel to the mid-axillary line of the trunk, and the movable arm directed toward the lateral condyle of the humerus, parallel to the midline of the humerus. For abduction, the fulcrum was placed over the acromion, the stationary arm parallel to the sternum and vertebral column, and the movable arm parallel to the anterior midline of the humerus. Internal and external rotation were measured with the shoulder in 90° of abduction and the elbow in 90° of flexion; the fulcrum was placed over the olecranon, the stationary arm parallel to the floor, and the movable arm aligned with the midline between the radius and ulna, parallel to the third metacarpal. For each direction, the mean of three consecutive goniometric readings was used for analysis. All range-of-motion measurements were performed by a single physiotherapist trained in standardized goniometric technique using the landmarks described above who was intended to remain blinded to group allocation and performed all goniometric measurements for all participants at both time points. No formal assessment of the success of assessor blinding was performed. Formal intra-rater reliability (e.g., intraclass correlation coefficient) was not calculated for the present sample; single-examiner goniometric measurement of shoulder range of motion using comparable standardized landmarks has previously been reported to show excellent intra-rater reliability (ICC approximately 0.91–0.99) [20]. Prospective calculation of intra-rater reliability is recommended for future trials.

### 2.7. Sample Size

No prospective sample-size calculation based on either registered co-primary outcome or a clinically meaningful between-group difference was performed. The available sample comprised 40 participants, with 20 participants allocated to each group. A retrospective sensitivity calculation conducted using G*Power version 3.1 indicated that, under the simplified assumptions of a two-tailed independent-groups comparison with α = 0.05 and 95% power, this sample size could detect a standardized between-group effect of approximately Cohen’s d = 1.17 [21,22]. This represents a very large effect. The sensitivity calculation does not establish that the sample was adequate for the registered co-primary outcomes, the fitted interaction models, clinically meaningful effects, or precise effect estimation. The trial should therefore be considered preliminary and exploratory, and the results should be interpreted primarily using raw baseline-adjusted between-group differences and their 95% confidence intervals rather than retrospective power or extreme standardized effect estimates.

### 2.8. Statistical Analysis

Data were analyzed using IBM SPSS Statistics version 26.0 (IBM Corp., Armonk, NY, USA). Additional HC3 robust regression, joint Wald testing, influence, leave-one-out, common-support, and graphical analyses were performed using Python 3.13 with statsmodels version 0.14.6, SciPy version 1.17.0, and Matplotlib version 3.10.8. Continuous variables are presented as mean ± standard deviation, and categorical variables as frequency and percentage. Baseline balance was assessed descriptively using signed standardized mean differences (SMDs; experimental minus control) with 95% confidence intervals. For continuous variables, SMDs were calculated by dividing the between-group mean difference by the pooled standard deviation; standardized differences in proportions were used for categorical variables. Confidence intervals were estimated using 10,000 group-stratified bootstrap resamples. Baseline significance tests were not used to evaluate the quality of randomization.

Inferential priority was assigned to the registered co-primary outcomes, the Shoulder Pain and Disability Index (SPADI) and Pittsburgh Sleep Quality Index (PSQI). Pain intensity measured using the Visual Analogue Scale (VAS) and shoulder flexion, abduction, internal rotation, and external rotation were considered secondary outcomes. For VAS and the range-of-motion outcomes, separate analysis of covariance (ANCOVA) models were fitted using the week-6 value as the dependent variable, treatment group as the fixed factor, and the corresponding baseline value as the covariate. Adjusted means, experimental-minus-control differences with 95% confidence intervals, F statistics, nominal *p* values, Holm-adjusted *p* values, and partial eta-squared (ηp^2^) were reported.

The homogeneity-of-regression-slopes assumption was evaluated using the group × baseline interaction. This assumption was satisfied for VAS and all range-of-motion outcomes. For SPADI and PSQI, significant group × baseline interactions indicated that the between-group difference varied according to baseline severity. Separate linear regression models were therefore fitted for SPADI and PSQI using the week-6 score as the dependent variable and treatment group, the mean-centered baseline score, and the group × baseline interaction as predictors. Treatment group was coded as 1 for the experimental group and 0 for the control group. HC3 heteroscedasticity-robust standard errors were used.

For each co-primary outcome, the confirmatory outcome-level null hypothesis was H0: βgroup = βgroup × baseline = 0. Within the fitted linear interaction model, this joint null corresponds to no between-group difference at any baseline value. The hypothesis was evaluated using a two-degree-of-freedom HC3 heteroscedasticity-robust Wald F test and reported as F(2, 36), together with its nominal and Holm-adjusted *p* values. The separate test of H0: βgroup × baseline = 0 was interpreted only as a test of heterogeneity of the between-group difference across baseline scores and was not used as the confirmatory outcome-level treatment test. Conditional experimental-minus-control differences were estimated at the 25th percentile, median, and 75th percentile of the observed baseline distribution to interpret the significant interactions. These percentile-specific contrasts were not treated as separate confirmatory hypotheses.

Model assumptions were evaluated using model-based diagnostics rather than the distributions of the raw outcome variables. Linearity was examined using outcome-versus-baseline plots within treatment groups and residual-versus-fitted plots. Homoscedasticity was evaluated using residual-versus-fitted plots and Breusch–Pagan tests, and residual normality was assessed using normal Q-Q plots and Shapiro–Wilk tests. Potentially influential observations were examined using externally studentized residuals, leverage values, and Cook’s distance. Because the co-primary interaction models were based on only 40 participants, leave-one-out analyses were performed to determine whether the joint outcome-level conclusions depended on any single participant.

To examine possible model extrapolation arising from baseline imbalance, group-specific minimum and maximum baseline values and the common-support interval shared by the two groups were calculated for each continuous baseline variable. Common support was defined descriptively as the intersection of the observed baseline ranges in the two groups. For SPADI and PSQI, the baseline values used for the 25th-percentile, median, and 75th-percentile conditional contrasts were compared with these common-support intervals. Sensitivity models additionally adjusted for baseline BMI and affected side. For VAS and the range-of-motion outcomes, the corresponding baseline outcome remained included as a covariate. For SPADI and PSQI, BMI and affected side were added while retaining treatment group, mean-centered baseline score, and the group × baseline interaction. The sensitivity analyses were used to assess the stability and direction of the estimates rather than to eliminate the possibility of residual confounding caused by chance baseline imbalance.

The principal outcome-level hypothesis family comprised seven tests: the two-degree-of-freedom joint tests for SPADI and PSQI and the baseline-adjusted group tests from the ANCOVA models for VAS and the four range-of-motion outcomes. The Holm step-down procedure was applied to the seven corresponding nominal *p* values to control the family-wise type I error rate at 0.05. Within-group comparisons, unadjusted change-score analyses, interaction-only tests, percentile-specific conditional contrasts, and additional sensitivity analyses were considered descriptive or supportive and were not included separately in the Holm-adjusted hypothesis family. All tests were two-tailed. Statistical significance for the seven principal outcome-level tests was determined using Holm-adjusted *p* values, whereas nominal *p* values for descriptive, conditional, diagnostic, or supportive analyses were reported for interpretation and were not considered independent confirmatory evidence.

## 3. Results

**Figure 1:**
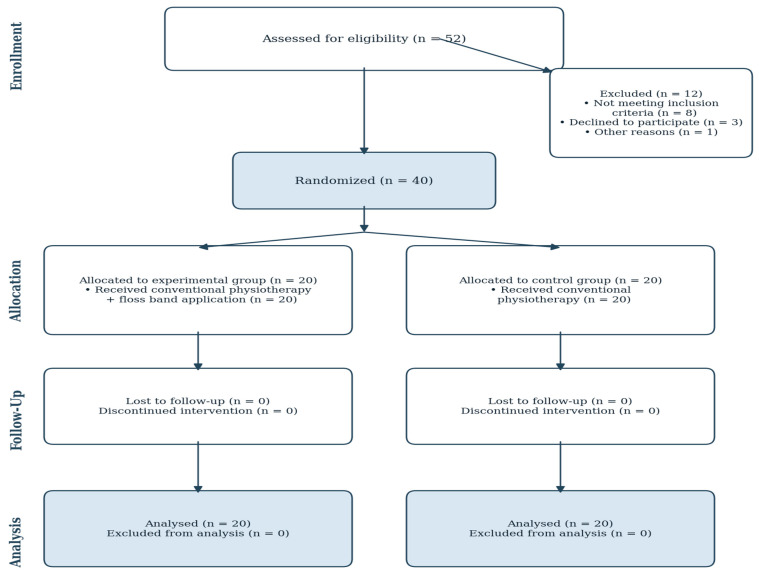
CONSORT flow diagram of participant enrolment, allocation, follow-up, and analysis.

Of 52 patients assessed for eligibility, 12 were excluded (8 did not meet the inclusion criteria, 3 declined to participate, and 1 for other reasons), leaving 40 who were randomized, 20 to the control group and 20 to the experimental group. All participants completed the 6-week intervention and both assessments, with no loss to follow-up; the flow of participants through the trial is shown in Figure 1. No participant discontinued treatment because of a safety concern, and no adverse skin, sensory, circulatory, or pain-related events requiring modification or termination of the intervention were observed or reported during the six-week treatment period. All randomized participants met the baseline pain criterion. Baseline VAS scores ranged from 6 to 9 in the experimental group and from 5 to 8 in the control group; therefore, no participant entered the analytical sample solely because of restricted shoulder range of motion. By design, all participants also had at least 90° of active shoulder flexion and abduction and a Modified Ashworth Scale score of 0. Available demographic, affected-side, pain, shoulder-mobility, SPADI, and PSQI characteristics are reported by group in Table 1. More detailed stroke-specific characteristics and HSP phenotypes were not systematically recorded.

Demographic and baseline clinical characteristics are presented in Table 1. Standardized mean differences indicated clinically relevant baseline imbalance in BMI (SMD = 0.80), affected-side distribution (absolute SMD = 0.81), VAS (SMD = 0.89), flexion (SMD = −0.69), abduction (SMD = −1.29), and internal rotation (SMD = −0.98). Smaller imbalances were also present for external rotation, SPADI, and PSQI. These differences were interpreted descriptively as chance imbalances and were addressed in the outcome-specific baseline-adjusted models.

Group-specific baseline ranges showed some common support for all continuous variables, although the extent of overlap varied. The common-support intervals were 23.0–31.1 kg/m^2^ for BMI, 6–8 points for VAS, 95–120° for flexion, 95–115° for abduction, 40–50° for internal rotation, 30–60° for external rotation, 45.38–68.46 points for SPADI, and 6–13 points for PSQI. The baseline values used for the SPADI conditional contrasts at the 25th percentile, median, and 75th percentile—54.61, 58.85, and 61.72 points—were within the shared SPADI range. The corresponding PSQI values of 8.00, 9.00, and 11.25 points were also within the shared PSQI range. Thus, the reported conditional contrasts did not require extrapolation beyond the baseline ranges shared by the two groups, although overlap was narrower for some range-of-motion outcomes.

Table 2 presents the descriptive pre- and post-treatment values and within-group mean changes observed over the six-week intervention period. These summaries are provided to describe the observed data and were not used to infer a between-group treatment effect. Because clinically relevant baseline imbalances were present, all between-group conclusions were based on the baseline-adjusted models reported in Table 3 and Table 4.

For VAS and all shoulder range-of-motion outcomes, the primary treatment effect was evaluated using the baseline-adjusted between-group difference at week 6. Because several clinical outcomes differed between the groups at baseline, each post-treatment outcome was analyzed using analysis of covariance (ANCOVA), with its corresponding baseline value included as a covariate. The homogeneity-of-regression-slopes assumption was satisfied for VAS and all shoulder range-of-motion outcomes, and the conventional baseline-adjusted ANCOVA results are presented in Table 3. The experimental group had more favorable baseline-adjusted post-treatment values for pain intensity, flexion, abduction, internal rotation, and external rotation (all *p* < 0.001). The baseline-adjusted differences were −2.33 points for VAS, +32.68° for flexion, +29.13° for abduction, +16.35° for internal rotation, and +15.95° for external rotation.

**Figure 2:**
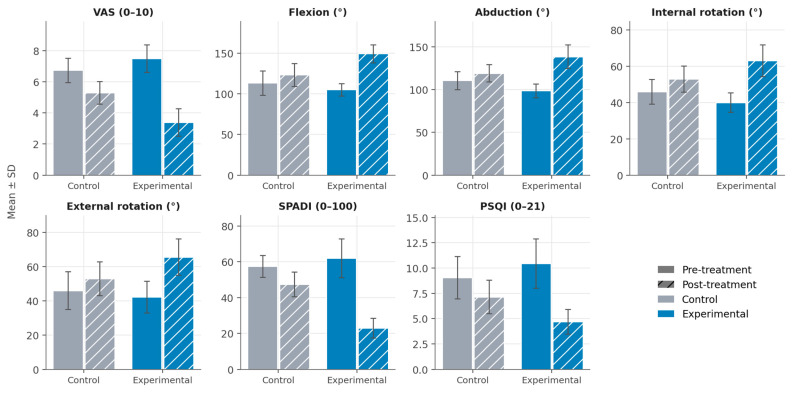
Mean (±standard deviation) pre- and post-treatment values for each outcome in the control and experimental groups. The figure presents unadjusted descriptive data; the corresponding inferential analyses are reported in Table 3 and Table 4.

For SPADI and PSQI, significant group × baseline interactions indicated that the experimental-minus-control difference varied according to baseline severity. The interaction coefficient was −1.051 for SPADI (HC3 SE = 0.186; 95% CI, −1.429 to −0.673; t(36) = −5.639; *p* = 2.11 × 10^−6^) and −0.427 for PSQI (HC3 SE = 0.115; 95% CI, −0.660 to −0.195; t(36) = −3.727; *p* = 6.63 × 10^−4^). These interaction-only tests describe effect heterogeneity and were not used as the confirmatory outcome-level treatment tests (Figure 2).

The confirmatory joint null hypothesis that both the group and group × baseline coefficients were zero was rejected for SPADI, F(2, 36) = 150.804, nominal *p* = 3.18 × 10^−18^, Holm-adjusted *p* = 2.22 × 10^−17^, and PSQI, F(2, 36) = 146.099, nominal *p* = 5.28 × 10^−18^, Holm-adjusted *p* = 3.17 × 10^−17^. Thus, within each fitted linear interaction model, the data were inconsistent with the absence of a between-group difference across the observed baseline range. The conditional experimental-minus-control differences were −20.87 points at the 25th percentile, −25.32 points at the median, and −28.34 points at the 75th percentile for SPADI. The corresponding PSQI differences were −2.48, −2.91, and −3.87 points. All conditional estimates favored the experimental group; however, these contrasts were used to interpret the significant interactions and were not entered separately into the Holm-adjusted hypothesis family. The five baseline-adjusted secondary-outcome group tests for VAS and shoulder flexion, abduction, internal rotation, and external rotation also remained statistically significant after Holm correction. Accordingly, the Holm-adjusted family comprised two joint co-primary outcome tests and five secondary-outcome group tests, rather than seven interaction or conditional treatment-effect tests.

Model diagnostics were examined because each interaction model was based on only 40 participants. For SPADI, the maximum absolute externally studentized residual was 2.63, maximum leverage was 0.312, and maximum Cook’s distance was 0.117. The Breusch–Pagan test did not provide strong evidence of heteroscedasticity (*p* = 0.096), and the Shapiro–Wilk test of residuals was not significant (*p* = 0.516). For PSQI, the corresponding diagnostic values were 3.71, 0.248, and 0.490. Evidence of heteroscedasticity was present for PSQI (Breusch–Pagan *p* = 0.002), supporting the use of HC3 robust inference, whereas the Shapiro–Wilk test was not statistically significant (*p* = 0.057). No observation had a Cook’s distance greater than 1. Across leave-one-out analyses, the HC3 robust joint Wald F statistic ranged from 110.54 to 168.30 for SPADI and from 132.07 to 164.83 for PSQI; all corresponding nominal *p* values remained below 1 × 10^−15^. The joint outcome-level conclusions therefore did not depend on any single participant. However, the relatively influential PSQI observation and the small sample indicate that the precise coefficients and interaction magnitudes should still be interpreted cautiously.

**Figure 3:**
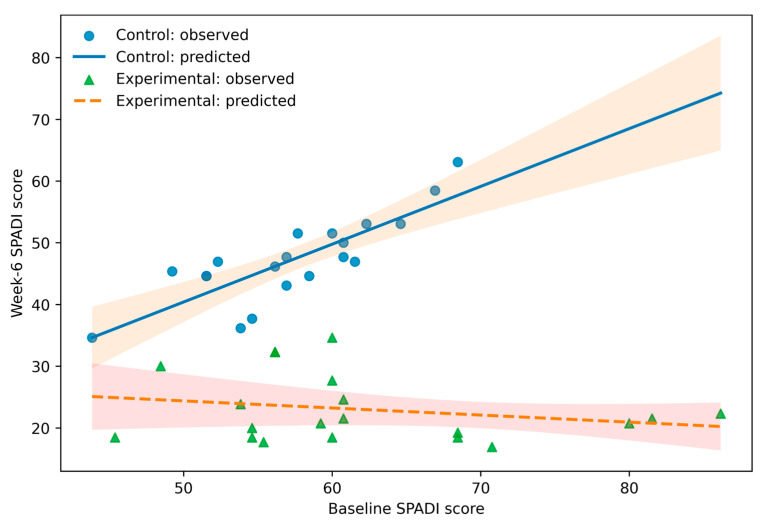
Predicted week-6 SPADI score across the observed baseline range. Model-predicted week-6 SPADI scores across the observed baseline SPADI range by treatment group. Lines represent predicted mean outcomes from the interaction model, shaded areas represent 95% confidence bands calculated using the HC3 robust covariance matrix, and points represent observed participant values.

**Figure 4:**
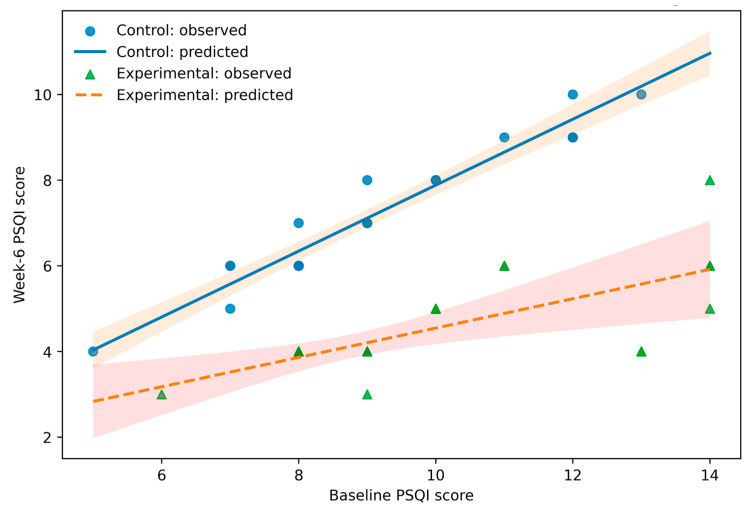
Predicted week-6 PSQI score across the observed baseline range. Model-predicted week-6 PSQI scores across the observed baseline PSQI range by treatment group. Lines represent predicted mean outcomes from the interaction model, shaded areas represent 95% confidence bands calculated using the HC3 robust covariance matrix, and points represent observed participant values.

Sensitivity analyses additionally adjusted for baseline BMI and affected side. For the secondary outcomes, the adjusted experimental-minus-control differences were −2.12 points for VAS (95% CI, −2.68 to −1.56; *p* = 5.43 × 10^−9^), +31.88° for flexion (95% CI, +24.60 to +39.15; *p* = 1.66 × 10^−10^), +28.32° for abduction (95% CI, +19.02 to +37.62; *p* = 4.45 × 10^−7^), +13.95° for internal rotation (95% CI, +9.75 to +18.15; *p* = 8.28 × 10^−8^), and +14.48° for external rotation (95% CI, +10.14 to +18.81; *p* = 7.42 × 10^−8^). For the co-primary outcomes, the BMI- and affected-side-adjusted HC3 robust joint tests remained significant for SPADI, F(2, 34) = 66.96, *p* = 1.62 × 10^−12^, and PSQI, F(2, 34) = 60.37, *p* = 6.49 × 10^−12^. The adjusted SPADI differences were −20.24 points at the 25th percentile (95% CI, −25.49 to −14.99), −24.70 points at the median (95% CI, −29.55 to −19.85), and −27.73 points at the 75th percentile (95% CI, −32.70 to −22.76). The corresponding PSQI differences were −2.36 points (95% CI, −2.88 to −1.83), −2.74 points (95% CI, −3.25 to −2.23), and −3.59 points (95% CI, −4.41 to −2.77). These sensitivity analyses supported the direction of the primary findings but did not eliminate uncertainty arising from chance imbalance, regression to the mean, or residual confounding (Figure 3 and Figure 4).

## 4. Discussion

This randomized controlled trial evaluated SPADI and PSQI as co-primary outcomes, with pain intensity and shoulder range of motion examined as secondary outcomes. Conventional physiotherapy plus a floss-band procedure with additional active shoulder movement was associated with greater short-term improvements than conventional physiotherapy alone. For pain intensity and range-of-motion outcomes, baseline-adjusted ANCOVA favored the experimental group. For SPADI and PSQI, significant group × baseline interactions indicated that the magnitude of the between-group difference varied according to baseline severity; nevertheless, conditional estimates at the lower quartile, median, and upper quartile of the observed baseline distributions consistently favored the experimental group. The two-degree-of-freedom joint tests of the group and group × baseline terms for SPADI and PSQI, together with the five baseline-adjusted secondary-outcome group tests, remained statistically significant after Holm correction. The interaction-only tests and percentile-specific contrasts were not treated as separate confirmatory treatment tests. The detailed modelling strategy was not prospectively specified in the registry; therefore, the adjusted, interaction, multiplicity, diagnostic, and sensitivity analyses should be interpreted as transparent analytical refinements supporting a preliminary trial rather than as components of a fully prespecified confirmatory analysis. Given the small sample and substantial baseline imbalance, interpretation should emphasize the raw baseline-adjusted between-group differences and their 95% confidence intervals rather than statistical significance or extreme standardized effects alone.

Importantly, the estimand was the incremental effect of the complete experimental procedure, which combined band compression, additional active shoulder movement, additional therapist interaction and treatment time, and intervention novelty. The design therefore did not isolate the physiological effect of tissue flossing or band compression, and the relative contributions of these components cannot be distinguished. Blinding applied only to the goniometric range-of-motion assessment. The co-primary SPADI and PSQI outcomes and the secondary VAS outcome were reported by participants who were aware of their treatment allocation. Consequently, these participant-reported findings were susceptible to detection and expectancy-related bias, particularly because the control condition did not match intervention novelty, additional attention, active movement, treatment time, or participant expectations. To our knowledge, this is the first controlled study to apply a floss-band procedure to the hemiplegic shoulder; previous evidence in stroke was limited to a single trial involving the ankle joint [12]. The present study therefore extends the preliminary application of tissue flossing to the paretic upper limb and evaluates the complete experimental procedure across pain, mobility, shoulder-related disability, and sleep quality within a clinical neurorehabilitation setting.

The large gains in shoulder range of motion observed after the complete experimental procedure are directionally consistent with the wider tissue-flossing literature, although the present design does not allow these gains to be attributed specifically to tissue flossing or band compression. A recent comprehensive systematic review and meta-analysis of randomized and crossover trials in healthy individuals found only a small, arguably trivial acute increase in range of motion after flossing and no significant pooled effect on pain, with most protocols consisting of a single, brief application [23]. In contrast, randomized trials that applied flossing over multiple sessions in symptomatic or mobility-restricted populations have reported clinically relevant gains: acute flossing improved ankle dorsiflexion in athletes with limited mobility [24], and a three-week, twice-weekly protocol combining flossing with manual therapy improved loaded dorsiflexion and selected mobility and balance outcomes in individuals with recurrent ankle sprains [25]. The discrepancy between the modest acute effects reported in healthy cohorts and the larger changes observed here may partly relate to the repeated six-week intervention, the additional active movement, and the presence of baseline pain and mobility limitation in a selected clinical sample. However, because HSP phenotypes and relevant stroke-specific characteristics were not systematically assessed, the observed response cannot be attributed specifically to soft-tissue restriction or to any other defined clinical mechanism.

Evidence specific to the upper limb supports the present results. In amateur overhead athletes, a single session of tissue flossing improved shoulder functional capacities to a degree comparable with instrument-assisted soft-tissue mobilization and kinesiology taping [26], indicating that the shoulder responds to flossing in a manner similar to the lower-limb joints that dominate the literature. The only previous application of a floss band in stroke, at the ankle, likewise produced improvements in dorsiflexion, balance, and gait [12]. The present findings do not establish that the mobility effects reported in previous flossing studies are attributable to tissue flossing at the hemiplegic shoulder. Rather, they show that a structured rehabilitation program incorporating band compression, additional active shoulder movement, and additional therapist interaction was associated with greater short-term mobility improvements than conventional physiotherapy alone.

Any mechanistic interpretation of the present findings is necessarily hypothesis-generating because no mechanistic outcome was measured and the intervention components were not experimentally separated. Several mechanical, vascular, and neurophysiological mechanisms have been proposed for floss-band application, including altered gliding between fascial layers, transient vascular occlusion followed by reactive hyperemia, and modulation of nociceptive and neuromuscular responses [27]. Indirect experimental evidence from a non-stroke population has shown that repeated flossing may influence the recovery of deep-fascia sliding and pressure pain threshold following exercise-induced muscle damage [28]. Such mechanisms may be relevant to some presentations of hemiplegic shoulder pain involving soft-tissue restriction or impaired tissue mobility. However, fascial mobility, local circulation, nociceptive sensitivity, muscle tone, shoulder subluxation, and the underlying pain phenotype were not assessed in the present study. Therefore, the observed clinical improvements cannot be attributed to fascial shearing, vascular reperfusion, neuromuscular modulation, tissue flossing, or any other specific physiological pathway. The proposed mechanisms should be regarded solely as hypotheses requiring direct investigation in trials using appropriate sham- or attention-and-exercise-matched comparators and mechanistic outcome measures. The clinical phenotype of the enrolled population also remains uncertain. Although all participants had affected-shoulder pain, preserved active elevation, and no clinically detectable upper-limb spasticity, stroke type, exact post-stroke duration, motor and sensory impairment, subluxation, neglect, limb dominance, and HSP phenotype were not systematically characterized. It is therefore not possible to determine whether the response was concentrated in a relatively homogeneous soft-tissue-restriction subgroup or occurred across different causes of post-stroke shoulder pain. The findings should not be generalized to the broader chronic HSP population.

The marked reduction in pain and shoulder disability observed after the complete experimental procedure is noteworthy when considered alongside adjunctive taping, one of the more extensively studied add-ons for hemiplegic shoulder pain. A randomized placebo-controlled trial of kinesiology taping added to conventional rehabilitation reported reductions in shoulder pain and subluxation and improvements in muscle activity and active range of motion [29], while a meta-analysis suggested modest benefits for upper-limb function after stroke [30]. However, heterogeneity in study designs, comparator conditions, and outcome measures limits firm conclusions regarding the specific efficacy of taping. Against this background, the consistent and large between-group differences across pain and range-of-motion outcomes in the present study are encouraging, while the absence of a significant pooled analgesic effect of flossing in healthy cohorts [23] suggests that the pain relief observed here may reflect the combined influence of a symptomatic population, repeated application, concurrent exercise, additional treatment time, and contextual effects rather than an intrinsic, population-independent analgesic action of the band.

Although the baseline-adjusted between-group differences were numerically large, their precision and likely magnitude in future samples remain uncertain. The observed within-group changes in pain and shoulder-related disability exceeded commonly cited minimal clinically important change thresholds derived from musculoskeletal pain and shoulder populations [15,31]. However, these thresholds were not established specifically for chronic hemiplegic shoulder pain and primarily concern within-patient change rather than baseline-adjusted between-group treatment effects. No prospective sample-size calculation was based on either co-primary outcome or on a clinically meaningful between-group difference. Therefore, the statistical significance and large standardized changes observed in this sample cannot be used retrospectively to establish adequate power or sample-size adequacy. Estimates from a small, single-center trial may be inflated by sampling variability, baseline imbalance, regression to the mean, measurement reactivity, and detection or expectancy-related bias. Interpretation should therefore focus on the raw baseline-adjusted between-group differences and their 95% confidence intervals. Extreme standardized change-score effects should not be interpreted as evidence of clinical importance, design adequacy, or the magnitude of effects likely to be observed in routine clinical practice. Replication in larger, prospectively powered, sham- or attention-and-exercise-matched trials is required to obtain more precise and stable estimates.

The improvement in sleep quality is, to our knowledge, a novel observation associated with a rehabilitation program incorporating a floss-band procedure and additional active shoulder movement. However, it cannot be attributed directly to tissue flossing, band compression, or any other individual component of the experimental intervention. The most probable explanation is indirect: the substantial reductions in pain and disability in the experimental group plausibly translated into better sleep, consistent with the well-recognized bidirectional relationship between pain and sleep [32], in which pain disturbs sleep and poor sleep may in turn increase pain sensitivity. This interpretation should be regarded as hypothesis-generating and warrants confirmation in studies designed specifically to examine sleep outcomes.

From a clinical perspective, the improvements observed in the control group indicate that the structured conventional physiotherapy program may itself be beneficial in chronic HSP. The greater short-term improvements observed with conventional physiotherapy plus a floss-band procedure with additional active shoulder movement provide preliminary support for further investigation of this complete combined approach in carefully selected patients with preserved active shoulder movement, no clinically detectable spasticity, and no contraindications to compression. However, because the experimental condition also included additional therapist interaction, treatment time, and intervention novelty, the findings do not demonstrate a specific clinical effect of tissue flossing or band compression. Accordingly, neither tissue flossing nor the complete procedure should currently be recommended as a routine component of post-stroke shoulder rehabilitation. Further adequately powered, sham- or attention-and-exercise-matched, assessor-blinded, multicenter trials involving more representative stroke populations and longer follow-up are required.

### Limitations

Several limitations should be acknowledged. No prospective sample-size calculation was performed using either registered co-primary outcome or a clinically meaningful between-group difference. The retrospective sensitivity calculation indicated only that the available sample could detect very large effects under simplified assumptions and did not establish adequate power or precision. The small sample also resulted in clinically relevant baseline imbalances in pain, shoulder mobility, BMI, and affected-side distribution. Although baseline-adjusted models, common-support assessments, influence diagnostics, leave-one-out analyses, and sensitivity models adjusting for BMI and affected side supported the direction of the findings, they cannot eliminate concerns regarding chance imbalance, regression to the mean, residual confounding, model dependence, or inflation of effect estimates. The significant group × baseline interactions for SPADI and PSQI further indicated that the between-group differences varied according to baseline severity. Accordingly, interpretation should emphasize the raw baseline-adjusted differences and their confidence intervals rather than statistical significance or extreme standardized effects.

Participants and the treating physiotherapist were aware of treatment allocation, and SPADI, PSQI, and VAS were reported by unblinded participants. Blinding was limited to the goniometric range-of-motion assessment, and successful maintenance of assessor blinding was not formally evaluated. The control condition was not matched for additional active movement, therapist interaction, treatment time, intervention novelty, credibility, or expectations, and treatment expectations were not measured. The observed differences may therefore reflect the complete experimental procedure and contextual or expectancy-related effects rather than a specific physiological effect of tissue flossing or band compression. Adverse events were monitored clinically but were not collected using a validated or independently adjudicated reporting system, so uncommon or delayed events may have been missed.

Generalizability is limited to a relatively young, higher-functioning, non-spastic subgroup with at least 90° of active shoulder flexion and abduction. Stroke type, exact time since stroke beyond six months, detailed motor and sensory impairment, glenohumeral subluxation, visuospatial neglect, limb dominance, and HSP phenotype were not systematically recorded. Consequently, it is not possible to determine whether the observed response was specific to a soft-tissue-restriction subgroup or occurred across clinically different causes of post-stroke shoulder pain. Outcomes were assessed only immediately after the six-week intervention, and the durability of the improvements remains unknown. Finally, within-group comparisons, unadjusted change-score summaries, interaction-only tests, percentile-specific contrasts, and additional sensitivity analyses were descriptive or supportive and were not treated as separate confirmatory evidence.

## 5. Conclusions

In this selected sample of adults aged 30–55 years with chronic affected-shoulder pain, preserved active shoulder elevation, and no clinically detectable upper-limb spasticity, adding a floss-band procedure with additional active shoulder movement to conventional physiotherapy was associated with greater short-term improvements than conventional physiotherapy alone. Baseline-adjusted analyses favored the experimental group for pain intensity and shoulder range of motion, while shoulder-related disability and sleep-quality outcomes favored the experimental group across the evaluated baseline levels, with differences varying according to baseline severity.

These findings represent the incremental effect of the complete experimental procedure, which combined band compression, additional active movement, therapist interaction, treatment time, and intervention novelty. The specific physiological contribution of tissue flossing or band compression therefore cannot be isolated. The results should be considered preliminary because of the small and imbalanced sample, absence of participant and therapist blinding, lack of a sham- or attention-and-exercise-matched control, and limited characterization of stroke-related impairment and HSP phenotypes. They should not be generalized to chronic HSP in general, to patients with greater motor impairment or spasticity, or to any specific shoulder-pain mechanism. Larger, prospectively powered, multicenter trials with matched comparator procedures, broader stroke populations, detailed clinical phenotyping, and longer follow-up are required before routine clinical recommendations can be made.

## Figures and Tables

**Table 1:** Demographic and baseline clinical characteristics with standardized mean differences between groups.

Variable	Control (*n* = 20)	Experimental (*n* = 20)	SMD (95% CI)
Age (years)	47.95 ± 4.75	48.65 ± 3.86	0.16 (−0.47, 0.77)
Sex—Female, *n* (%)	10 (50)	8 (40)	−0.20 (−0.89, 0.43)
Sex—Male, *n* (%)	10 (50)	12 (60)	-
BMI (kg/m^2^)	25.05 ± 3.23	27.33 ± 2.42	0.80 (0.19, 1.57)
Marital status—Married, *n* (%)	15 (75)	18 (90)	0.40 (−0.25, 1.04)
Marital status—Single, *n* (%)	5 (25)	2 (10)	-
Education—Primary/Secondary, *n* (%)	10 (50)	14 (70)	0.42 (−0.21, 1.16)
Education—High school, *n* (%)	5 (25)	2 (10)	−0.40 (−1.04, 0.23)
Education—University+, *n* (%)	5 (25)	4 (20)	−0.12 (−0.81, 0.52)
Affected side—Right, *n* (%)	19 (95)	13 (65)	−0.81 (−1.45, −0.23)
Affected side—Left, *n* (%)	1 (5)	7 (35)	-
**Baseline clinical outcomes (mean ± SD)**			
VAS	6.75 ± 0.79	7.5 ± 0.89	0.89 (0.31, 1.57)
Flexion (°)	113.5 ± 14.96	105.25 ± 7.69	−0.69 (−1.42, −0.10)
Abduction (°)	110.75 ± 10.55	98.75 ± 7.93	−1.29 (−2.13, −0.69)
Internal rotation (°)	46 ± 6.81	40 ± 5.38	−0.98 (−1.57, −0.47)
External rotation (°)	46 ± 11.07	42.25 ± 9.24	−0.37 (−1.01, 0.24)
SPADI	57.42 ± 6.09	62.03 ± 10.81	0.53 (−0.07, 1.10)
PSQI	9.05 ± 2.09	10.45 ± 2.44	0.62 (0.02, 1.30)

Values are presented as mean ± standard deviation or *n* (%). SMDs are signed as experimental minus control. For continuous variables, positive values indicate higher baseline means in the experimental group; for categorical variables, positive values indicate a higher proportion in the category shown. Values closer to zero indicate better baseline balance. Confidence intervals were obtained using 10,000 group-stratified bootstrap resamples. SMD: standardized mean difference; CI: confidence interval; BMI: body mass index; VAS: Visual Analogue Scale; SPADI: Shoulder Pain and Disability Index; PSQI: Pittsburgh Sleep Quality Index. All participants had a baseline VAS score of ≥3; none was enrolled solely on the basis of restricted range of motion. Stroke type, exact time since stroke, detailed motor or sensory impairment, glenohumeral subluxation, visuospatial neglect, limb dominance, and HSP phenotype were not systematically recorded.

**Table 2:** Descriptive baseline, week-6, and change values in the control and experimental groups.

Outcome	Group	Baseline	Post-Treatment	Change, Week 6 − Baseline (95% CI)	Within-Group *p* Value
SPADI	Control	57.42 ± 6.09	47.34 ± 6.9	10.08 (8.25, 11.91)	<0.001
	Experimental	62.03 ± 10.81	23 ± 5.47	39.04 (32.87, 45.2)	<0.001
PSQI	Control	9.05 ± 2.09	7.15 ± 1.66	1.9 (1.6, 2.2)	<0.001
	Experimental	10.45 ± 2.44	4.7 ± 1.22	5.75 (4.89, 6.61)	<0.001
VAS	Control	6.75 ± 0.79	5.3 ± 0.73	1.45 (1.21, 1.69)	<0.001
	Experimental	7.5 ± 0.89	3.4 ± 0.88	4.1 (3.67, 4.53)	<0.001
Flexion (°)	Control	113.5 ± 14.96	123.5 ± 14.24	−10 (−12.01, −7.99)	<0.001
	Experimental	105.25 ± 7.69	149.5 ± 10.99	−44.25 (−49.58, −38.92)	<0.001
Abduction (°)	Control	110.75 ± 10.55	119.25 ± 10.04	−8.5 (−10.21, −6.79)	<0.001
	Experimental	98.75 ± 7.93	138.6 ± 13.87	−39.85 (−45.95, −33.75)	<0.001
Internal rotation (°)	Control	46 ± 6.81	53 ± 7.15	−7 (−8.4, −5.6)	<0.001
	Experimental	40 ± 5.38	63.1 ± 8.68	−23.1 (−25.9, −20.3)	<0.001
External rotation (°)	Control	46 ± 11.07	52.9 ± 9.88	−6.9 (−8.35, −5.45)	<0.001
	Experimental	42.25 ± 9.24	65.6 ± 10.65	−23.35 (−26.61, −20.09)	<0.001

Paired-samples *t*-test was used for within-group comparisons. CI: confidence interval; The mean difference is reported as in the source data: for VAS, SPADI, and PSQI, a positive value indicates improvement through a reduction in score, whereas for range-of-motion variables, improvement corresponds to an increase from pre- to post-treatment.

**Table 3:** Baseline-adjusted between-group comparisons for outcomes satisfying the homogeneity-of-regression-slopes assumption.

Outcome	Adjusted Mean Control	Adjusted Mean Experimental	Adjusted Mean Difference (95% CI)	F(1, 37)	Partial η^2^	Nominal *p* Value	Holm-Adjusted *p* Value
VAS	5.52	3.18	−2.33 (−2.80, −1.87)	104.02	0.74	2.67 × 10^−12^	1.07 × 10^−11^
Flexion (°)	120.16	152.84	+32.68 (+26.96, +38.41)	133.92	0.78	7.41 × 10^−14^	3.71 × 10^−13^
Abduction (°)	114.36	143.49	+29.13 (+21.81, +36.46)	64.91	0.64	1.17 × 10^−9^	1.17 × 10^−9^
Internal rotation (°)	49.87	66.23	+16.35 (+12.92, +19.79)	93.06	0.72	1.21 × 10^−11^	3.63 × 10^−11^
External rotation (°)	51.27	67.23	+15.95 (+12.50, +19.41)	87.69	0.70	2.66 × 10^−11^	5.33 × 10^−11^

ANCOVA was performed with the post-treatment value as the dependent variable, treatment group as the fixed factor, and the corresponding baseline value as the covariate. Adjusted means were estimated at the pooled baseline mean. Between-group differences were calculated as experimental minus control. A negative difference favors the experimental group for VAS, whereas a positive difference favors the experimental group for range-of-motion outcomes. The Holm step-down procedure was applied across the seven principal outcome-level tests. CI: confidence interval; VAS: Visual Analogue Scale.

**Table 4:** (A,B) Complete HC3 robust interaction-model results, joint outcome-level tests, and baseline-dependent conditional contrasts for SPADI and PSQI.

Panel A. Complete interaction-model coefficients and confirmatory joint tests							
Outcome	Model term	β	HC3 SE	95% CI	Test statistic	Nominal *p* value	Holm-adjusted *p* value
SPADI	Intercept	49.503	0.971	47.534 to 51.472	t(36) = 50.991	3.63 × 10^−35^	—
SPADI	Experimental group	−26.243	1.700	−29.691 to −22.795	t(36) = −15.435	1.89 × 10^−17^	—
SPADI	Mean-centered baseline	0.936	0.160	0.612 to 1.261	t(36) = 5.847	1.11 × 10^−6^	—
SPADI	Group × baseline	−1.051	0.186	−1.429 to −0.673	t(36) = −5.639	2.11 × 10^−6^	—
SPADI	Joint group + interaction test	—	—	—	F(2, 36) = 150.804	3.18 × 10^−18^	2.22 × 10^−17^
PSQI	Intercept	7.689	0.108	7.469 to 7.908	t(36) = 71.043	2.66 × 10^−40^	—
PSQI	Experimental group	−3.229	0.199	−3.633 to −2.824	t(36) = −16.192	4.17 × 10^−18^	—
PSQI	Mean-centered baseline	0.770	0.047	0.675 to 0.864	t(36) = 16.548	2.09 × 10^−18^	—
PSQI	Group × baseline	−0.427	0.115	−0.660 to −0.195	t(36) = −3.727	6.63 × 10^−4^	—
PSQI	Joint group + interaction test	—	—	—	F(2, 36) = 146.099	5.28 × 10^−18^	3.17 × 10^−17^
Panel B. Conditional experimental-minus-control contrasts							
Outcome	Baseline level	Baseline score		Experimental−control difference (95% CI)		t(36)	Nominal *p* value
SPADI	P25	54.61		−20.87 (−25.06, −16.67)		−10.097	4.80 × 10^−12^
SPADI	Median	58.85		−25.32 (−28.83, −21.80)		−14.619	1.03 × 10^−16^
SPADI	P75	61.72		−28.34 (−31.76, −24.92)		−16.800	1.29 × 10^−18^
PSQI	P25	8.00		−2.48 (−2.88, −2.08)		−12.656	8.10 × 10^−15^
PSQI	Median	9.00		−2.91 (−3.26, −2.56)		−16.977	9.18 × 10^−19^
PSQI	P75	11.25		−3.87 (−4.53, −3.21)		−11.946	4.38 × 10^−14^

Separate linear regression models were fitted for SPADI and PSQI with the week-6 score as the dependent variable and treatment group, mean-centered baseline score, and the group × baseline interaction as predictors. HC3 heteroscedasticity-robust standard errors were used. The confirmatory outcome-level test jointly evaluated H_0_: βgroup = βgroup × baseline = 0 using an HC3 robust Wald F test with 2 and 36 degrees of freedom. The interaction-only *p* value evaluates heterogeneity of the between-group difference and was not used as the confirmatory treatment test. Percentile-specific contrasts were used to interpret the interaction and were not included separately in the Holm family. The experimental-group coefficient represents the experimental-minus-control difference at the pooled mean baseline score because baseline was mean-centered. CI: confidence interval; P25: 25th percentile; P75: 75th percentile.

## Data Availability

The original contributions presented in this study are included in the article. Further inquiries can be directed to the corresponding author.
